# Development of a village health worker care model to reduce cardiovascular risk in areas of armed conflict: Qualitative results from a participatory planning process in Myanmar

**DOI:** 10.1371/journal.pgph.0005610

**Published:** 2026-11-06

**Authors:** Eindra Htoo, Parveen Parmar, Adam Richards, Tara Russell, Catherine Lee, Saw Sha Kler Law, Saw Kyaw Myint, Zay Yar Phyo Aung, Tom Traill

**Affiliations:** 1 Author affiliation withheld for security reasons; 2 Department of Emergency Medicine, Harbor-UCLA Medical Center, Torrance, California, United States of America; 3 Department of Emergency Medicine, University of California, Los Angeles, California, United States of America; 4 Center for Humanitarian Health, Department of International Health, Johns Hopkins Bloomberg School of Public Health, Baltimore, Maryland, United States of America; 5 ASEAN Institute for Health Development, Mahidol University, Bangkok, Thailand; 6 Department of Health, Karen Department of Health and Welfare, Karen State, Myanmar; University of Gothenburg: Goteborgs Universitet, SWEDEN

## Abstract

Cardiovascular disease (CVD) is the leading cause of death in Myanmar, but care models are lacking to deliver evidence-based treatments to reduce CVD risks in areas of armed conflict. Village health workers (VHWs) play a key role in bridging communities and clinics, but their role in CVD prevention and management in conflict-affected settings has not been clearly defined. This study aimed to describe the system for CVD care and to elicit perspectives on how VHWs might contribute to a village-based care model to overcome structural and behavioral barriers to CVD risk factor control in remote rural areas experiencing active armed conflict in Karen State, Myanmar. We conducted 33 semi-structured key informant interviews with patients, VHWs, clinic staff, ethnic health organization leaders, donors, and non-government organization health experts. Content analysis of participant responses suggested many people were unaware of CVD and its risks, and there were persistent gaps in CVD screening, medication adherence, and referral systems. Transportation costs and hazards, medicine shortages, and security risks made it difficult for patients to visit clinics or achieve continuity of care. Respondents believed VHWs could play an important role in improving CVD care by providing health education sessions, screening for high blood pressure and diabetes, delivering medications and supporting adherence, and facilitating referrals. Respondents reported that expanding VHW roles could make CVD care more accessible, particularly where travel to clinics was unsafe or unaffordable. Respondents perceived that VHWs have the potential to address CVD care gaps, and that targeted training and regular supervision of VHWs, coupled with structured systems for referral and follow-up, could enhance continuity of care, adherence to treatment, and community resilience to acute conflict events.

## Introduction

Cardiovascular disease (CVD) is the leading cause of death and disability in many fragile and conflict-affected states, including Myanmar [1,2]. However, the humanitarian system has neglected to invest in CVD diagnosis and treatment; one study estimated that non-communicable diseases (NCDs), which include CVD, received only 0.44% of overseas development aid for health targeted to internally displaced persons (IDPs) globally [3]. The proportion of health aid specifically reaching Ethnic Health Organization (EHO) services in the study setting are not available. Coverage of interventions proven to reduce CVD risk is low, and evidence is lacking to support the effectiveness of care models to diagnose and treat hypertension, diabetes, and elevated CVD risk (with statins) in areas of active conflict [3–5].

Karen State, located in Eastern Myanmar along the border with Thailand, exemplifies this global pattern of underinvestment and low service coverage for CVD risk factors. A military coup in 2021 acutely exacerbated access to CVD services for over 260,000 IDPs [6] who, after six decades of chronic armed conflict, have come to rely on EHOs for most health services [7,8]. Health financing is dependent on donors who have prioritized maternal and child health and infectious diseases such as malaria and tuberculosis over NCDs. Resources for CVD risk factor management are limited, and coverage is low [9].

Following the 2021 military coup, many healthcare workers joined the nationwide Civil Disobedience Movement (CDM), refusing to work under the military-controlled health system. Some CDM-affiliated healthcare professionals subsequently relocated to conflict-affected and ethnic-controlled areas, where they contributed to alternative and community-based health services, including through collaboration with existing ethnic and community-based health organizations. Although these health workers have helped expand clinical capacity in some areas, their work has been constrained by insecurity, limited resources, and threats to healthcare personnel and facilities [10].

In 2022, the Karen Department of Health and Welfare (KDHW) was funded to conduct a three-phase study to develop and evaluate a village health worker (VHW) care model to increase access to CVD services in remote rural areas of Karen State experiencing violence due to armed conflict. Phase 1 focused on formative research and participatory development of the VHW care model, including the qualitative interviews reported in this manuscript. The resulting care model was subsequently assessed for feasibility and acceptability in three villages before being evaluated in a cluster randomized controlled trial (cRCT) examining its impact on medication adherence, CVD risk, and cost-effectiveness [11,12].

KDHW chose to develop a VHW-centered care model based on their successful experience with decentralized approaches to maternity care and malaria control, delivered by maternal health and village malaria workers [13–15]. By bringing services to beneficiaries instead of requiring beneficiaries to travel to services, decentralized models can reduce transportation costs and other structural barriers to care and promote health equity [16]. Local service provision has the potential to reduce exposure to military checkpoints by reducing the need to travel through militarized areas to access care [17,18]. Multiple studies support the effectiveness of community health worker (CHW) care models to control hypertension and other CVD risk factors in low and middle-income countries (LMICs), but their potential contribution to CVD prevention and management in conflict-affected areas has not been extensively explored [19–27]. A global priority-setting exercise ranked developing and testing the effectiveness of CHW care models as the top priority for research on cardio-metabolic syndrome in humanitarian emergencies [28].

Here we present findings from the qualitative interviews conducted as part of Phase 1 to inform development of the VHW care model subsequently assessed and evaluated in later study phases. The qualitative aims were to describe the system for CVD care and elicit perspectives on how VHWs might contribute to a village-based care model to overcome structural and behavioral barriers to CVD risk factor control in remote rural areas experiencing active armed conflict. We describe in the methods section below how stakeholder perspectives were used to seed a causal loop diagram of the complex adaptive system of CVD care and to co-create a VHW care model to be tested in a type 2 hybrid effectiveness-implementation study anchored in a cRCT.

## Methods

### Ethics statement

This study was deemed exempt by the University of Southern California Office of Human Subjects Research and by The George Washington University Committee on Human Research (IRB# NCR234977); it received ethical approval from the regional Community Ethics Advisory Board (CEAB-2022–009), which is a community-led group that provides ethical guidance and oversight for research. Funding was provided by Research for Health in Humanitarian Crises (R2HC/Elrha #78698) and a Research Innovation Award from the Office of Research Excellence, Milken Institute School of Public Health, George Washington University (#09RIA042022). All semi-structured KII respondents provided informed verbal consent.

### Study design

This study was guided by a participatory, iterative development process to capture the complexity of healthcare for CVD risk management in a conflict-affected community health system, reflecting the perspectives of multiple stakeholders. This approach was informed by systems thinking tools to understand how interventions may impact a complex, interconnected system. We looked particularly at Causal Loop Analysis (CLA) - an approach that has been effectively applied in diverse health contexts to uncover underlying dynamics governing service delivery and outcomes, including CVD care models for refugees [29,30]. CLA is a visual method used to examine how multiple factors interact within a complex system. It highlights feedback loops and interconnections, helping to identify barriers, reinforcing patterns, and potential leverage points for improving care [31]. However, participation in the planned CLA workshop was constrained by some study participants’ availability and competing priorities during the worsening conflict. Consequently, it was not possible to conduct a confirmatory workshop to finalize the causal loop diagram (Fig 1) with participant stakeholders. In spite of this limitation, the authors were able to use the diagram development process and thematic analysis to demonstrate VHWs as critical leverage points for improving cardiovascular outcomes, informing interventions aimed at strengthening the system of community-based support for CVD care delivery and medication adherence [31].

**Fig 1:**
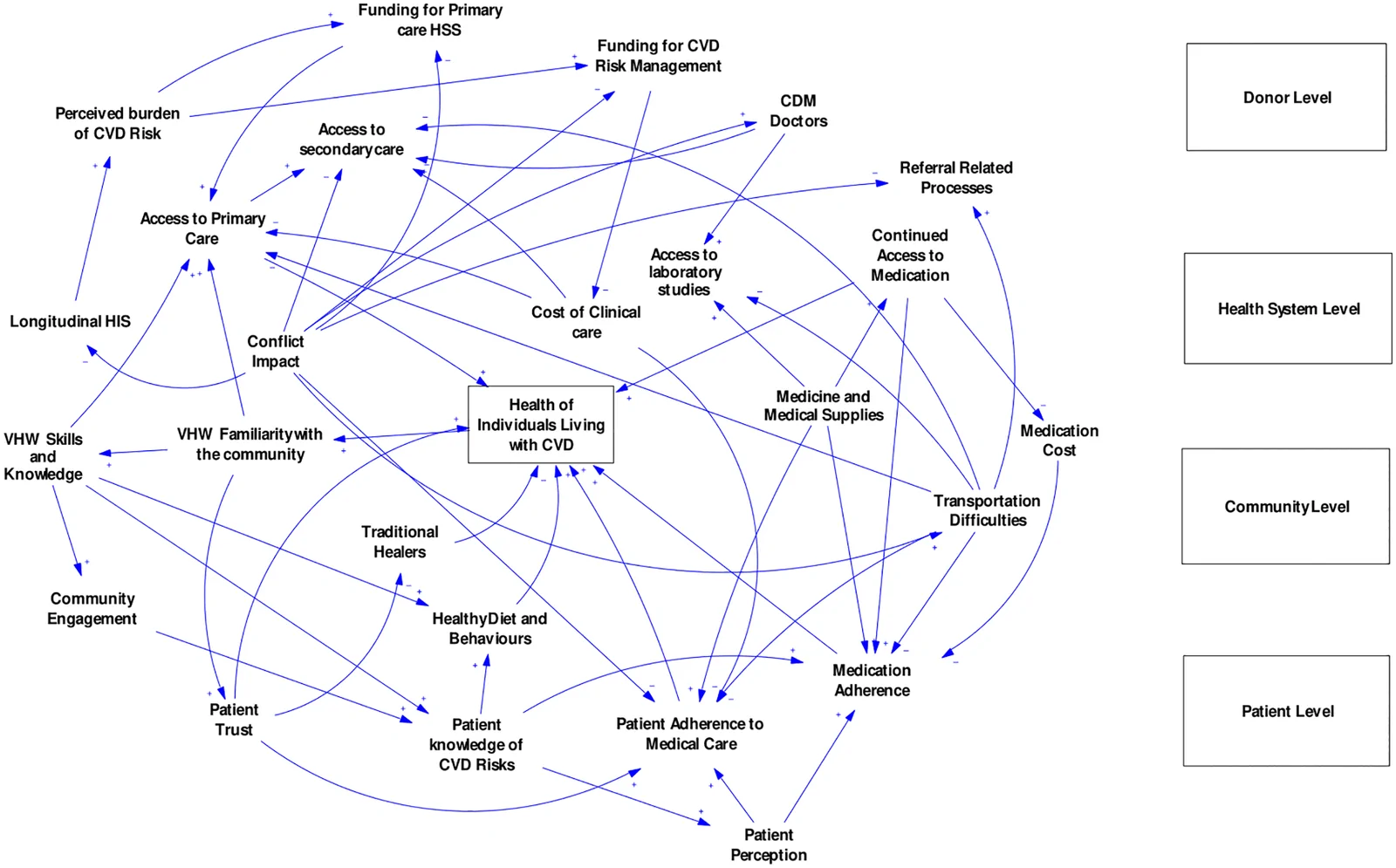
First draft of causal loop diagram.

VHWs are a cadre of CHWs in the KDHW system of care who are from the village where they work [19]. They receive a brief training covering prevention, basic treatment and referral for endemic diseases, and a modest monthly stipend. Depending on the specific health project responsibilities and workload, VHWs may receive additional training and payments, for example for malaria control in endemic areas. Most VHWs have additional responsibilities and sources of income outside the health system*,* such as seasonal farm work, kitchen gardens, or small market stalls.

The team developed four semi-structured interview guides (S1 Text to S4 Text), tailored to the four stakeholder levels: patient, provider, health-system, and donor levels, to explore factors influencing CVD care and the delivery of NCD and CVD care within the broader healthcare system, and to inform the design of new CVD program activities with VHWs. The guide drew on Parveen Parmer’s (PP) prior experience with co-creation of a causal loop diagram for CVD care of refugees in Jordan [30], as well as the health behavior framework and the chronic care model [32,33]. The premise of the health behavior framework is that in order to influence health behaviors that are complex and multi-dimensional, such as participation in screening for CVD risk factors, it is necessary to design interventions that address multiple drivers of the behavior at patient, provider, health system, and financing levels. The chronic care model is an effective framework for taskshifting when supported by multi-component delivery that improves care processes and outcomes while reducing costs for chronic conditions, including diabetes and hypertension [34].

### Participant selection and recruitment

Participants were selected to include multiple perspectives from a diverse range of stakeholders and roles in CVD care, while balancing the exigencies of an ambitious timeline to transcribe, analyze, and present preliminary findings in the participatory workshop. Recruitment began on 25 November 2022 and was completed on 7 April 2023. The initial purposive sampling strategy aimed to recruit 38 participants: 12 patients with CVD, 4 VHWs, 6 EHO clinic staff, 8 EHO leaders, 8 donors and Non-government Organizations (NGOs) with NCD experience. Preference was given to individuals with direct experience or expertise relevant to CVD care and the community health system [31]. Participants were initially approached by email or face-to-face and provided with information about the study and an invitation to participate in a key informant interview (KII). The final sample consisted of 33 participants: 12 patients, 4 VHWs, 6 EHO clinic staff/medics, 7 EHO leaders, and 4 donors/NGO representatives (see Table 1). Recruitment of donors and NGO representatives was lower than initially planned due to scheduling challenges during the short recruitment period between ethical approval and the CLA workshop.

**Table 1:** Individuals interviewed and domains of semi-structured interviews.

Interviewee type	Domains explored	Number of interviewees
Patients	Health-seeking behavior, understanding of CVD, medication adherence, barriers to care, experience with clinics	12
VHWs	Community engagement, role in screening and follow-up, and challenges in service delivery	4
Clinic Staff and Medics	CVD treatment protocols, training needs, supply availability, patient monitoring, referral processes	6
EHO Leadership	Health system structure, service planning, integration with broader care systems, priorities for NCD care	7
Donors and NGO Representatives	Funding priorities, support for NCD/CVD programs, and coordination challenges	4

### Data collection

All interviews were carried out in the respondent’s preferred language (English, Burmese, or Karen) and both in-person and virtual interviews across Thailand and Eastern Myanmar according to their availability. Eindra Htoo (EH), Parveen Parmar (PP), Adam Richards (AR), and EHO staff conducted the interviews. EHO interviewers are experienced public health professionals, with experience in interviewing and data collection, and knowledge of the local context and security conditions. KII participants knew their interviewer, or were familiar with their role and motivations, as a result of their organizational association. Participants were provided with further information through the consent process. Participants gave verbal consent, and interviews were audio-recorded, then transcribed and, where necessary, translated into English for analysis. Each KII lasted approximately 45 minutes to one hour. The recorded files and transcripts were kept secure and only accessible by study team members. In order to assure safety in an atmosphere of heightened security tensions, names were not recorded. For clarity, roles were noted on each transcript (i.e., EHO leadership, VHW, etc.).

### Data analysis

A first-stage rapid analysis was conducted by PP, with a starting framework provided by the draft causal loop diagram, while being open to new concepts and themes provided by respondents. This initial analysis had to be carried out under time constraints due to security and other field realities. Based on this first analysis, the themes were then organized by the investigators into a preliminary diagram of the health system as it relates to care for CVD risk. This initial analysis informed a written policy brief that was shared with a broad group of stakeholders. Following this, the study team conducted a more formal and in-depth thematic analysis of the KII transcripts to refine preliminary results [35]. PP and EH coded the data manually using an Excel matrix. Where there were discrepancies between these code sets, the two coders reviewed the data excerpts and developed a consensus on the most appropriate interpretation (S1 Table). This was later reviewed and revised with reference to the translated transcripts by Tara Russell (TR) and EH as the themes were developed. Other members of the research team contributed to reviewing and interpreting the findings, refining the identified themes, and contextualizing the findings within the local health system and the development of the VHW care model. Individual author contributions are further detailed in the Author Contributions statement and COREQ Checklist (S1 Checklist). The diagram was revised and reformatted in Canva [36], presenting the identified themes within the health systems as enablers or challenges (or both) in advancing CVD prevention and management in this context.

## Results

We conducted 33 KIIs. The following section describes the trust, health behavior, health seeking, access, and challenges in CVD delivery at each level of the community health system.

The following illustrative diagram (Fig 2) summarizes the themes, identifying those that are challenges or enabling factors affecting the implementation of VHW-led care for individuals living with CVD across patient, provider, health system, and donor levels. The codebook with illustrative quotes for each theme is available in S1 Table.

**Fig 2:**
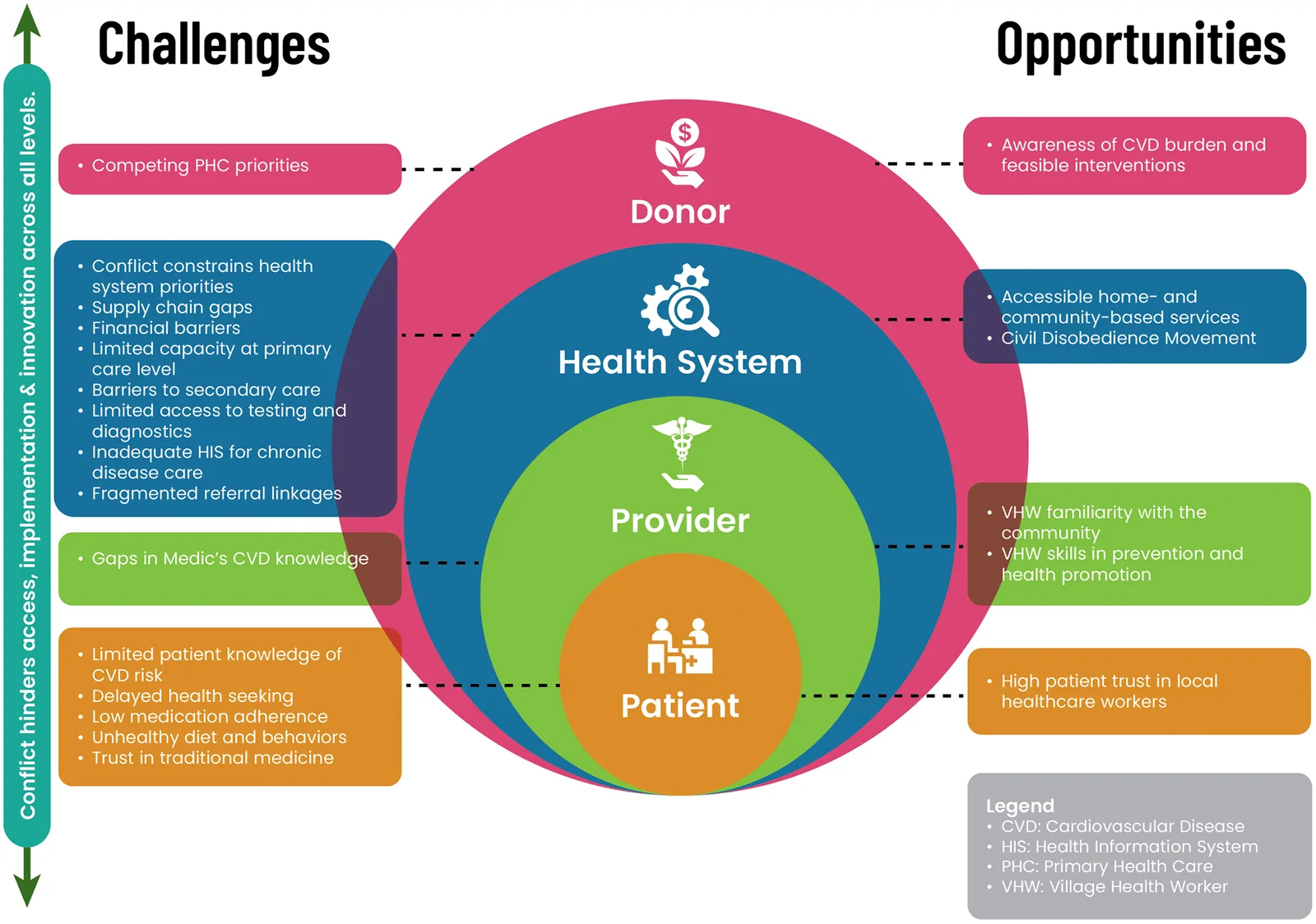
Challenges and opportunities for VHW care models to reduce CVD risk in areas of armed conflict.

Across patient, provider, health system, and donor levels, conflict-related factors compounded existing challenges to CVD care. Respondents described the conflict leading to clinic closures, staff and patients fleeing for their safety, and transportation became increasingly risky. Security threats, checkpoints, and rising costs disrupted service delivery, supply chains, and affected the accessibility of care, especially for those patients needing referral or chronic disease care. These findings illustrate how conflict interacted with existing financial, geographic, and health-system barriers to continuity of CVD care.

### Patient level

#### High patient trust in local healthcare workers.

In EHO clinics and villages, trust in local healthcare workers was widespread across Karen communities. EHO staff noted that VHWs were uniquely positioned as trusted community members, who could speak with patients about their concerns, and monitor medicine adherence “*without barriers*”. Patients mentioned that they sought care at these clinics due to positive experiences and their confidence in the staff.

*“They [local health workers] have a good attitude. I always come here for my treatment.” CVD Patient*

#### Limited patient knowledge of CVD risk.

Many individuals lacked basic awareness of CVD and its long-term complications. VHWs and clinic staff mentioned that sharing information among community members could help to spread knowledge about CVD risks and management, with several EHO staff and leadership mentioning that VHWs could provide “*health education if we train them*”.

*“Because of the outreach activity, because of health education and community engagement activities, [community members] start to hear about the diseases, and they know they have got the information and also from one person to another person that they will share the information.” EHO Staff*

Strategies such as VHW-facilitated interactive education sessions, videos, and social group counseling were proposed to enhance patient engagement. Education about the consequences of untreated hypertension and diabetes was also perceived as important to promote medication adherence and manage chronic diseases effectively.

#### Delayed health seeking.

Timely health seeking was impacted by each patient’s perception of CVD. The perception that CVD was less critical than other endemic diseases made patients less likely to prioritize CVD care, particularly when they faced other concerns in the protracted and worsening conflict, such as financial hardship, safety threats, or transportation challenges. Since CVD is often initially asymptomatic, patients had further reasons not to consider CVD as an urgent or serious threat, and to delay care-seeking. An interviewed EHO program officer described this as “They do not care because those diseases may not harm them for the first few years”.

#### Low medication adherence.

Some KII respondents were concerned about potential problems with medication adherence. Clinic staff report that many patients stop taking medication once they feel better, and only a few patients follow their treatment consistently. One EHO advisor noted that if treatment could be provided, patients would “*need supervision by one of their family member[s] or a volunteer health worker.*”

One VHW also stated, “*They [the patients] only go back to the clinics when the diseases reappear again”.* Adherence to medical advice was also influenced by patients’ perceptions of CVD and constrained by logistics and financial barriers, as well as limited awareness and understanding of the chronic nature of CVD and the importance of continued treatment even when symptoms were absent or had improved.*”*

#### Unhealthy diet and behaviors.

Although health workers routinely provided diet and behavior advice, they believed that VHWs could be provided with additional training to provide dedicated follow-up with patients. Health workers reported concerns that diets relied heavily on oily and salty foods, as well as high salt dietary and traditional medicine supplements They also reported that limited local food diversity and difficult living conditions hampered lifestyle changes. Smoking, alcohol, and betel nut use remained common. One program administrator described this:

*“I think the main factor is their stressful living conditions. They have been living in conflict zones along the border since the country’s independence in 1948 (...) This has resulted in a substandard lifestyle where fish paste is consumed more than protein-rich meat.” NGO health program administrator*

#### Trust in traditional medicine.

Traditional herbal remedies play a prominent role in service areas. Traditional medicine includes home preparations of herbs and roots, commercially available powders, as well as services provided by traditional healers that include ceremonies, injections, and oral preparations. Several interviewees reported that some patients will use herbal or traditional remedies before symptoms become severe, when other medicine isn’t available, or after trying western medicine and not observing any improvement.

*“I took modern medicine but did not feel better. So, I took traditional medicine and medical plants and roots. After taking them, I got better.” CVD Patient*

EHO workers reported that these issues need to be treated with sensitivity, and VHW’s trusted position could be utilized to redirect patients to evidence-based treatments: *“if we [medics] give the explanation, they will just scold us or they won’t accept it.”*

### Provider level

#### VHW familiarity with the community.

Participants felt that VHWs who are recruited from communities have unique knowledge and close relationships with the communities they serve. In the view of one NGO program administrator, they “*can help organize them without having any social barriers.”* Others noted that VHWs can serve as a bridge between clinics and remote populations, catalyzing initial engagement with the health system and sustaining continuity of care. In times of heightened conflict when mobile teams “*delayed reaching those places on schedule*”, as members of this community, VHWs remain in the village or in displacement sites and can provide a supportive role (EHO staff).

#### VHW skills in prevention and health promotion.

Moreover, VHW skills and knowledge were important for effective chronic disease management.

Respondents suggested that VHWs could provide essential services such as home visits, blood pressure and glucose measurement, and health education, if they “*were trained and provided with the necessary medical supplies”*. VHW respondents described their existing roles managing patient information, assisting medics, monitoring medication adherence, and organizing village health initiatives. However, VHWs were not typically trained in CVD care, limiting their ability to support management of hypertension and diabetes.

#### Gaps in Medic’s CVD knowledge.

“*In primary health care…the highest rank is medic. The medic curriculum includes CVD, and they know how to treat but not as good as a doctor. It's not to blame them. It’s their capacity.” EHO Staff*

Participants described how some medics have extensive experience and training in CVD risk factor management, including lung and heart function, lifestyle changes and prescription of appropriate medications. Many had been trained on protocols from the Burma Border Guidelines, Maternal and Child Health, or Integrated Management of Childhood Illness guidelines. However, some clinical guidelines for CVD were incomplete or outdated, and many providers lack opportunities to practice knowledge gained from didactic training in a mentored and supervised visit with real patients. Clinic staff frequently mentioned that both medics and VHWs require updated, focused training to provide evidence-based care to reduce risk. EHO staff and leadership acknowledged that VHWs would also need supportive supervision, as well as additional remuneration, if they were to take on more responsibilities.

*“If we are going to assign additional tasks to the VHWs, they should receive extra support, or they may not feel motivated to participate.” Clinic staff*

### Health system level

#### Conflict constrains health system priorities.

The current acute phases of chronic conflict had severely strained health system supply chains and human resources.

*“The military coup has had a significant impact on healthcare in the region, leading to delays in the transportation of medical supplies, difficulties in accessing advanced healthcare facilities in towns, and security concerns due to the presence of multiple checkpoints.” EHO Clinic Staff*

EHO decision-makers and health workers themselves acknowledged that threats to the safety of workers and their families can take precedence over VHW-related activities:

*“When they get information that the airstrike is coming this way or to this area, the other junta soldiers are burning the house, [the health workers] have to run (…) In this situation, we [as their leaders] can’t do anything. We can’t blame them “Why aren’t you treating the patient? Why aren’t you measuring blood pressure? (…) it's affecting providing medical services and I think there's no answer for that.” EHO coordinator*

#### Supply chain gaps.

Availability and continuity of medicine supplies were major challenges across clinics, reported by both health workers and patients. The lack of blood pressure cuffs and glucometers limited providers’ ability to conduct proper diagnoses. Medication stockouts were common, leading some clinics to instruct patients to buy from local pharmacies. Therefore, maintaining continued access to medications was a significant concern for both providers and patients, particularly when conflict interrupts supply chains. When patients are diagnosed with a chronic disease, they are initially prescribed monthly dosages, which is extended to two-three months as symptoms stabilize. Giving medications for two to three months is more convenient for both providers and patients, but careful monitoring is needed to ensure patient follow-up and adherence monitoring, which could be provided by VHWs.

*“The clinic staff initially prescribed a monthly dosage. However, now that they trust me to take my medicines regularly, I am receiving a three-month dosage. They always check if I have any medicine left from the previous dosage.” CVD Patient*

#### Financial barriers.

*“Inflation since the military coup has made it difficult for many of us to afford the medications we need. I could not afford it if I had to buy the monthly medicines I have to take.” CVD Patient*

Medications are ostensibly available free of charge from KDHW clinics, but some CVD patients seek care from other providers, and several stakeholders described substantial financial barriers to care and medication. Many patients could not afford to complete full courses of treatment, often relying on sharing prescriptions or buying small amounts at a time. While the cost of diagnosis and consultation is generally fee-free for most EHO clinics, some patients may donate or pay a nominal fee. While this is not expected from all patients, some respondents believed this could be a barrier for some low-income patients. Some, but not all, Thailand-based clinics provide limited fee-free secondary care to alleviate this.

For acute and complex cases, the cost of transportation to higher-level care, particularly for cross-border referrals to Thai clinics, could be prohibitively expensive. Transportation costs for some referrals might be covered based on the patient's symptoms and the availability of facilities at the referral center; however, coverage of such costs is conditional on clinic cash reserves, donor support, and often depends on time- or geographically- limited projects.

“*Some patients…can afford to pay the transportation charges themselves. For those who could not pay for it, we pay the cost with the clinic’s funds for patient referrals. I think there is a limit for the transportation expenses per patient.*” *EHO clinic staff*

#### Accessible home- and community-based services.

Respondents believed village-level services for screening (e.g., screening among the community for diabetes, hypertension, obesity, or smoking habits), home-based treatment follow-up, and linking them to appropriate care facilities were effective strategies. Some respondents suggested that mobile outreach activities could be beneficial, but these require trained volunteers and strong monitoring systems.

#### Limited capacity at primary care level.

One of the major challenges for clinics to manage CVD was the lack of trained professionals, with staff not trained for complicated cases. Some EHO leaders pointed out challenges such as physical access in the monsoon season, basic infrastructure, limited supplies, and low awareness of services, which continued to limit access and discourage clinic attendance:

*“People prefer to go to clinics in towns because they believe that they have access to all necessary tools and medical supplies. This [village] clinic provides primary healthcare, so it's understandable that they may not have access to many tools and medical supplies.” CVD Patient*

#### Access barriers to secondary care.

Guidelines recommend that complicated cases with signs and symptoms of acute stroke or heart attack be referred to hospitals for confirmation of diagnosis and specialized treatment. In KDHW service areas they operated four secondary care facilities, one of which “*is often subject to artillery fire, forcing patients and medical staff to flee to other villages and temporarily take shelter in school buildings*.” *EHO Staff*

Travel can be difficult, and dangerous:

“*During the rainy season, the trip is also dangerous as we have to cross a waterway, and there are security concerns as well*.” *Patient*

Travelling to secondary care facilities outside the KDHW system sometimes involved crossing the border into Thailand. Physical and financial barriers to travel already highlighted are exacerbated by restrictions on patients to travel to Thailand.

“*They are being caught by the police because they don't have legal documentation and this is also an effect [on care-seeking]*.” *EHO staff*

#### Civil Disobedience Movement (CDM).

During KIIs with the EHO leaders and donors, they stated that healthcare workers who fled government crackdowns have helped to fill gaps in secondary care since the coup.

*“These [new] sorts of secondary facilities [with CDM doctors] could be good places for referral. They can manage the higher-level advanced case.” Donor*

CDM professionals contribute to mobile medical units, provide training, clinical supervision, and identify CVD risk factors in the community. Nonetheless, ongoing, locally based continuous assistance, including medicine availability, is required to enhance their effectiveness. It is also not clear how long recently arrived clinicians, many of whom previously lived in urban centers of central Myanmar, will remain in remote rural border areas.

#### Limited access to testing and diagnostics.

While some clinics have basic diagnostic tools like malaria microscopy, many clinics experienced what one EHO staff member described as *“numerous insufficiencies”* in basic equipment and capacity. Patients need to travel to the nearby city if they need even basic laboratory tests (e.g., serum creatinine or lipid profile). Supply chain challenges and equipment shortages hinder effective diagnostics. Some EHO leaders from the KIIs suggested expanding access to diagnostic tools and offering more frequent training to strengthen capacity. They also suggested that VHWs could be trained to use blood pressure cuffs and glucometers for monitoring.

#### Inadequate Health Information System (HIS) for chronic disease care.

Medics recorded clinical information using paper records and Excel spreadsheets that made it difficult, if not impossible, to view information from prior visits. Monthly reports were submitted online to the headquarters. However, some clinic staff stated that due to mass displacement, and the sensitivity of population data and geolocation information of populated areas, population statistics are not reliable. Data entry errors and a lack of standardized guidelines for follow-up tracking caused challenges in decision-making and program planning for leadership.

EHO staff complained that they had tried new technology solutions, but each one had introduced new problems. One app displayed continuous “*network errors*”, another failed because providers had misunderstood when to use it, a third tool couldn’t track patients from one visit to the next, meaning health workers couldn’t “*assess the blood pressure and glucose records*”. Individual patient booklets were frequently lost by patients. On the other hand, EHO workers believed that VHWs could collect and record reliable data from patients, as their position of trust in the community increased the acceptability of regular data collection from patients.

#### Fragmented referral linkages.

Referral processes were fragmented and increasingly difficult after the coup. According to KIIs with patients, they faced language barriers, safety risks, rising costs, and travel restrictions. These restrictions forced changes in the referral process, and patients were reluctant to travel. Some clinics stopped providing documentation to the patient to take with them to their referral, with one health worker recalling: “*In the past, we provided them with a referral letter, but we are no longer providing it due to security concerns.”* The lack of clear EHO referral protocols also impacted the quality and efficiency of referrals. Suggestions from staff and patients included improving referral systems, increasing staff capacity, ensuring better coordination with other providers, and developing clinic-based systems for tracking follow-up and medication adherence.

### Donor level

#### Competing priorities: infectious diseases, crisis response and primary health care service packages.

Participants reported that while the burden of CVD has risen, the resources allocated to addressing it remain very low. According to EHO leaders, CVD services largely relied on referral to other service providers in Thailand. Most funding to Eastern Myanmar continued to prioritize endemic communicable diseases, which were perceived as more urgent and locally manageable. Chronic conditions like CVD, which require long-term management and investment, received comparatively less attention, especially in active conflict settings.

Participants noted the dependence of community health systems on international funding. Modest local financing exists, but individuals have very little capacity to pay out-of-pocket for care. The lack of user fees reduces financial burden on patients, but also reduces resources available for local funding of local priorities. The lack of a defined tax base or revenue model in local areas raised concerns about the sustainability of healthcare financing. Even though some clinics benefited from volunteer and CDM doctors, these opportunities were fragile and required additional investment in supplies, medications, and capacity building to be sustained.

*“There will be challenges for the organizations to fund long-term treatment and the patients will also have financial issues as he/she will have to long-stay at a place far from home. It would be challenging to provide such healthcare services for NCD patients due to its chronic nature.” NGO Leadership*

Participants reported that Health system strengthening (HSS) and primary health care (PHC) projects have gained donors’ interest, particularly around improving basic health service delivery and governance among local communities. However, HSS and PHC funding has focused on communicable diseases and maternal and child health. Although service packages nominally cover the cost for anti-hypertensive medications, both procurement and utilization remain low.

Moreover, the ongoing crisis of COVID and the Coup have increased donor investment in “*emergency humanitarian support*”, that often includes food and basic supplies for cooking and shelter.

*“We were very busy with the COVID response and a [then the] coup happened (….) we try to shift focus more on this emergency response (...) so, people are very busy and overloaded with these ongoing conflicts and also system strengthening focus. And also, I think [this is] also the donor’s perspective. So, the NCD was overlooked.” Donor*

#### Awareness of CVD burden and feasible interventions.

*“We are quite sure that mortality rates are increasing…although there is no concrete statistic, up-to-date statistics we are quite sure that we still need to focus on these and maternal and child health” Donor*

Although concern about CVD was increasing locally, the donors’ perception was that it took time to see outcomes from chronic care provision, and results were difficult to monitor. Donors suggested that to receive more support for NCD care, it would be important to raise awareness using data and advocacy, and by linking CVD efforts with broader health system improvements.

## Discussion

This study describes qualitative findings from stakeholder interviews to inform development of the first VHW-led intervention to improve screening and access to care for CVD in a conflict-affected region of Myanmar. Previous studies have described development of care models for refugee populations, but not IDPs or those living in active conflict zones [30,37]. Our study extends this work to explore possible roles for VHWs in active conflict zones [4,5,38]. In the context of this project, we have consulted with individuals with a stake in CVD care in an area of Eastern Myanmar with protracted and escalating conflict. Our final analysis and care model highlight local strengths and opportunities of taking a VHW-led approach.

Findings presented here contributed to a workshop to develop a VHW care model that involved universal screening of adults 40 years and older for CVD risk factors, and enrollment of eligible villagers in monthly household visits by the VHW. This model subsequently was shown to be acceptable and feasible to implement in three villages [11]; a cRCT subsequently tested the impact of the care model on medication adherence, CVD risk and cost effectiveness [12].

Based on the results of the analysis, the authors identified five potential areas of influence for VHWs, as well as associated challenges in this context. The first four hold relevance to both conflict and stable settings; the fifth describes the unique role VHWs can play in active conflict zones through their consistent presence in communities, even during times of acute crisis or relocation.

### 1. VHW-led community-based prevention via risk factor modification

This study suggests that VHWs are uniquely positioned to deliver culturally tailored interventions that promote risk factor modification, an approach supported by multiple studies highlighting the effectiveness of VHWs in NCD prevention [20–22,30,39,40]. Low awareness of CVD among patients and health workers was a critical barrier according to the study findings. Respondents agreed that VHWs might fill this awareness gap by conducting regular health education sessions, promoting smoking cessation, supporting healthy diet and physical exercises, and emphasizing early screening and continued medical adherence.

Transportation barriers and the current ongoing conflict limit the ability of villagers to access clinic-based care, underscoring the importance of VHW-led outreach. Through home visits and community education sessions, study respondents believed that VHWs can raise awareness of long-term CVD risks, emphasize preventive measures, and link community members to clinic resources. This multifaceted approach has proven effective in low-resource settings where healthcare systems function as complex adaptive entities requiring locally driven strategies [20,41,42], and respondents feel that VHWs can play similar roles in war-torn areas.

### 2. VHW screening of communities for elevated CVD risk, diabetes, and hypertension

Many community members only seek care when symptoms become severe, often missing the window for early intervention. Critics of community screening for hypertension note that some programs suffer from inaccurate blood pressure assessment, low rates of follow-up, and treatment adherence and are unlikely to improve population-level blood pressure control. Participants in our study were more optimistic about the potential for active community screening to lower CVD risk in a population with low awareness of CVD risk factors. They believed that, if such screening were conducted by trusted community members and coupled with active household follow-up, it would obviate the need for expensive and potentially unsafe travel to distant clinics. Participants perceived that VHWs can play a similar role to their contributions to infectious diseases control and reproductive health service access: they can serve as the initial contact, identify at-risk individuals and facilitate referrals. In addition, for chronic conditions VHWs can also contribute to longitudinal disease care, as noted below. Participant perceptions align with an increasing body of evidence that supports the effectiveness of screening programs for hypertension and other CVD risk factors when screening is coupled with tailored follow-up by community health workers [21,22,39,43–46].

### 3. VHW support for adherence to medication, disease management, and clinical care

Low medication adherence came out as an important concern, with patients often discontinuing treatment once they feel better, or due to financial constraints, or due to a lack of understanding of the chronic nature of CVD. Costs for accessing care and medicine supply chain challenges are exacerbated by political instability and displacement [47]. Moreover, when clinics close or staff relocate due to active conflict, patients may lose contact with their regular healthcare providers. In these unstable conditions, some respondents feared that incorrect information on CVD and treatment spreads more easily, which may reinforce poor adherence and delayed health seeking.

According to stakeholders, with additional basic training VHWs can also provide monthly monitoring of blood pressure and (when applicable) blood glucose. By building on to the existing reporting pathways, they can then report their findings to the regional medic on a monthly basis for review. This emphasizes the importance of frontline agents (like VHWs) to improve the reach and timeliness of care in conflict-affected regions [44].

Study respondents in supervisory and leadership roles believe that VHWs can provide vital support for adherence via home visits and consistent follow-up by reminding patients about dosing, clarifying side effects, and reinforcing the importance of continuous medication, even when symptoms subside. Previous research has shown that VHW interventions can improve medication adherence for chronic conditions in LMICs [11,20,23,42,46]. Moreover, VHWs can quickly relay shortages or clinical concerns back to EHOs, ensuring that gaps in healthcare are addressed.

### 4. Standardized referral pathways (From Clinic) and follow-up (To Clinic)

The study highlights major gaps in the referral system for CVD patients in Eastern Myanmar. Providers reported that financial constraints, transportation difficulties, and security risks limit access to advanced care. Moreover, the lack of standardized referral guidelines and follow-up mechanisms makes patient referrals challenging. Border restrictions, high costs, and poor coordination hinder access, similar to other studies [30,47]. Even when referrals occur, tracking patient outcomes is still difficult.

To improve this, experienced providers in our KII suggested a standardized referral system could set clear criteria for CVD cases, safe referral pathways, and systematic tracking for follow-ups. Strengthening VHW roles in post-referral support through home visits and digital health tracking could improve adherence and ensure better long-term management [11,48].

### 5. Responsive to an evolving context

Subsequent to the period of data collection for this study, additional challenges to chronic disease care in Eastern Myanmar have emerged, which we acknowledge here and will inform future planning. The conflict in Southeastern Myanmar has evolved, with increasing air strikes in the region. Myanmar experiences some of the highest levels of attacks on healthcare workers and facilities [49]. Arguably, the role of VHWs, who may relocate with their fellow villagers when forced to relocate, remains more essential than ever. Despite many challenges, such as limited resources, insecure environments, and shifting donor priorities toward humanitarian and communicable disease programs, VHWs can fill the gaps in screening, medication adherence, and support health behavior adoption. VHWs also provide a low-cost means of extending clinical care in a shrinking funding environment. At the same time, solutions must also appropriately support VHWs, with salaries that recognize the additional responsibilities and specific expertise, and with appropriate levels of clinical oversight, supervision and duty of care [50].

With the worsening insecurity, VHWs may also have a role in supporting patients to manage mental health symptoms. Conflict-affected populations face chronic stressors such as displacement, ongoing violence, and economic hardship, which can worsen both mental health conditions and physical health, like hypertension or diabetes. Several respondents in our study mentioned that fear, stress, and financial worries made it harder for patients to manage their health and continue taking medicines. Clinic Staff also mentioned that they had concerns with medication adherence due to these unstable conditions. Entrenched beliefs about the severity of CVD and the efficacy of medicines may discourage patients from seeking help, further complicating CVD management [51,52]. Evidence suggests training health workers in health education and mental health can help address these barriers to care, and can improve treatment adherence and outcomes [53], and promote a more integrated approach to healthcare [53,54]. A recent study in a Thai camp for Myanmar refugees found that a mental health intervention delivered by trained community members substantially improved mental health symptoms and modestly increased in adherence to CVD medications [55].

The feasibility and desirability of a VHW model for NCDs described here builds on increasing evidence of the effectiveness and efficiency of CHWs to reduce CVD risk in more stable settings [21,22,39,40,56–58], and reflects the optimism of study participants that similar benefits might extend to CHW care models to reduce CVD in areas of active armed conflict. Several crucial aspects of task-shifting responsibility to hyper-local health workforce were spontaneously mentioned by respondents and deserve additional comment. For example, the present study did not explicitly revisit tensions related to health worker mental health, motivation, burnout and remuneration that are nearly universal across CHW programs; we previously document substantial unmet needs for mental health support for VHWs and remuneration that was too low to support families, leading to attrition [59,60]. The VHW care model tested in subsequent phases of this study [11,12] raised the CHW stipend to the maximum possible value, while balancing the distortion of payment scales across the KDHW health workforce.

The present study also did not explicitly revisit ethical implications related to security risks for personal and family safety when tasks are shifted to the lowest paid health workers who live in villages experiencing armed conflict. Local actors in Southeast Myanmar have decades of experience monitoring and managing security risks for the humanitarian health workforce composed of individuals with varying degrees of training and experience, the large majority of whom lack a medical or nursing degree. KDHW and other EHOs have trained many hundreds of VHWs for programs targeting conditions other than NCDs, and as noted by respondents the health system accepts that it is entirely appropriate for VHWs to prioritize personal and family safety – similar to any other villager. During intervention-development workshops KDHW and the research team reflected on how years of collaborative work and study during a prolonged, chronic emergency has led to the general belief that by decoupling care from facilities, decentralized VHW care models may decrease security risk for the population as a whole, compared to a counterfactual scenario under which every villager must travel individually – and, often, accompanied by family members – on roads past unpredictable and perilous checkpoints, or hike over forested hills along landmine-laden paths, in order to access care. The VHW care model evaluated in subsequent phases of the study recruited individuals who were already VHWs, and any additional security risks for them were likely to be marginal; the number of additional trips to clinic was limited and most of their personal risk of violence resulted from their residence in a village at risk of aerial bombardment or soldier attack. The caveats above should not diminish the imperative to acknowledge that the elevation of VHWs and expansion of their roles may carry increased risk. The authors believe this calls for greater advocacy for VHWs, CHWs and other health workers to be adequately paid, supervised and supplied, with opportunities to seek mental health support, receive alerts from early warning systems, and resources to facilitate relocation when required by insecurity. We more directly explored these themes in qualitative interviews conducted to assess implementation science domains as part of the subsequent type 1 hybrid effectiveness-implementation study testing the VHW care model developed from the formative work reported here.

## 6. Strength of the study

The approach taken in this study promoted the voices of all relevant stakeholders (from CVD patients to VHWs to the institutions providing health services and donors, but heavily weighted to the community level). It therefore grounded the model that was tested in the needs and feasibility of working in a setting in which operationalization of interventions is a central concern. It has shaped the acceptance and engagement with the study following this initial stage, which continues beyond the type 1 hybrid effectiveness-implementation science study until today.

### Limitations of the study

Patients, VHWs, and other clinical staff interviewed for this study were residents in remote rural areas of Eastern Myanmar served by a single EHO, which may limit generalizability to other areas of Myanmar or to armed conflicts in other regions.

Some community members and VHWs may not have felt comfortable engaging fully, cautious of topics perceived to be sensitive to funding decisions, or a more general need to be circumspect when discussing the details of service delivery in an active conflict zone. Communication and transportation barriers, worsened by active conflict, made several interviews challenging.

Due to worsening security during this period, it was not possible to have a final workshop to confirm with stakeholders the complex care system represented by the draft causal loop diagram. In spite of this limitation, the authors were able to use the draft diagram and thematic analysis to demonstrate that the strengths of VHWs can be leveraged to improve cardiovascular outcomes, informing interventions aimed at strengthening community-based support for CVD care adherence and delivery [31].

## Conclusion

This qualitative study underscores the feasibility, capacity and willingness of local stakeholders to develop and test VHW care models to mitigate the growing burden of CVD in conflict-affected Eastern Myanmar. Study respondents endorsed the capacity of VHWs to play multiple constructive roles in CVD management for populations confronting dual burdens of chronic disease and conflict.

Our study may hold implications for other academic-agency research partnerships interested in employing similar qualitative methods to inform the co-creation of care models designed to reduce CVD risk in acute crises. It can provide a blueprint for the inclusive design of such programs, though advocacy is still required to increase global allocations of aid to NCDs and to the adequate salary and support to be given to VHWs in such settings.

## Supporting information

S1 Table Codebook. (DOCX)

S1 Checklist COREQ checklist. (DOCX)

S1 Text Qualitative interview guide, EHO leadership, program managers. (DOCX)

S2 Text EHO clinic leadership qualitative interview guide and facility assessment tool. (DOCX)

S3 Text Donor/NGO representative qualitative interview guide. (DOCX)

S4 Text Qualitative Guide: Patient/VHW qualitative interview guide. (DOCX)
