# Supplementary material for: Development of a village health worker care model to reduce cardiovascular risk in areas of armed conflict: Qualitative results from a participatory planning process in Myanmar
Source: PLOS Glob Public Health. 2026 Nov 6;6(11):e0005610. doi: 10.1371/journal.pgph.0005610 (PMC13743020; doi:10.1371/journal.pgph.0005610)
Supplement: Supplementary file 1 [file pgph.0005610.s001.docx]

**S1 Table: Codebook**

| Level | Theme | Definition | Illustrative quote | Type |
| --- | --- | --- | --- | --- |
| Patient | Health, Diet and Behaviors | On CVD and its risk factors | They do not care unless the cases are urgent... likewise, they prefer taking medicine when they are sick. | Deductive |
|  | Patient knowledge of CVD Risk | On CVD and its risk factors | I'm sure that the people need certain level of knowledge and understanding for this type of cardiovascular disease, especially these are connected to each other, people are not aware. | Deductive |
|  | Patient Adherence to Longitudinal Care for CVD Risk | Patient views/behaviors in engaging in long term care for CVD risk. | It is not because of the conflict, but because of their attitude. They only seek care when they were experiencing signs and symptoms. | Deductive |
|  | Medication Adherence | To CVD treatment | I'm worried about the drug compliance thing because if they are really stressed, you know, in this conflict area, people are really stressed | Deductive |
|  | Patient Trust | Relating to patient trust in medical care. | The medical staffs are very kind to the patients, and I believe that their treatments and quality of care are good, although I am not entirely sure. I simply take the medication they prescribe. They treat the patients nicely. | Deductive |
|  | Patient perceptions | Care and treatment | The clinic is near enough and everything is fine. They just stop visiting the clinic. But I always visit and get my medicines. | Inductive |
| Community level | Traditional healers and medicine | Relating to non-allopathic medical care. | Some patients seek medical help from local individuals who administer injections, while others rely on herbal remedies. Personally, I usually come to this clinic and take my medications regularly | Deductive |
|  | Transportation | Related to transportation of patients to receive clinical care. | They are day workers, it is difficult for them to travel. They are being caught by the polices because they don't have a legal documentation and this is also an effect. | Deductive |
|  | VHW Familiarity and Community | Relating to VHW relationship with the community. | Each village will hire one staff member to assist in making connections with clinic staff. One CHW in charge of communities can go there and provides medicine as well. At the moment, no health worker has fled due to conflict. | Deductive |
|  | VHW abilities and knowledge |  | They can manage doing diabetes and hypertension related tasks like taking blood samples for malaria and diabetes, measuring blood pressure. | Deductive |
|  | Community Screening |  | Screening and monitoring of blood pressure levels for people over 40 years old would also be beneficial, and the assistance of VHWs would be valuable. | Inductive |
| Health System | Availability of Supplies (medications, BP Cuffs, etc.) | Availability of CVD supplies/medications in the clinics. | We always have a BP cuff ready at our clinic. But it is challenging when the clinic runs out of medications sometimes. At such time, we have to prescribe the available medication instead, such as Amlodipine if we run out of Hydrochlorothiazide, and Atenolol if we run out of Amlodipine. | Deductive |
|  | Access to Medications | Continuous access to medications for treatment of CVD. | At the initial visit of a hypertension patient, we don’t usually provide a monthly dosage, but instead we provide a week dosage for people from nearby villages and two-week dosage for patients from distant villages, and then schedule their next appointment | Deductive |
|  | Medication Cost | Any costs incurred by patient’s related to medications | Some patients purchase their medicines at drug stores instead of getting them at the clinic. | Deductive |
|  | Cost of Clinical Care | Any costs incurred by patients in obtaining clinical care (does not include transportation/medications). | At the clinic from Kyondoe township, I have to pay the fee of charges for the treatment and there are other costs as well. If my blood pressure and glucose level were too high, they referred me to a hospital from the township. The total cost for charges is 30,000 MMK or 80,000MMK, or 100,000 MMK for only treatment without including the transportation and meal expenses. | Deductive |
|  | Medic/CHW Knowledge-Abilities | Relating to Medic or CHW knowledge on CVD risk identification and treatment. | All the staffs at this clinic and other clinics treat the patients with respect. The perform history taking and examination. Some people go the clinic in Shanywathit. They are sometimes referred to Hlaingbwe hospital. As this clinic (Htee Tha Blu Hta clinic) is quite small, some patients are referred to other hospitals if the clinic staffs has no ability to provide require treatement.. At the clinic, the staffs measure the body weight and blood pressure. Some staffs (medics/clinic in charge) perform measurements such as checking blood vessels by pressing them and listening through the stethoscope, etc. His assistances do not perform physical examination. | Deductive |
|  | HR challenges |  | To provide advanced services like echocardiogram, angiogram and ECG, they need to provide trainings for their technicians, nurses, and doctors. If there are fewer trained staffs available, it may become more challenging to offer these services. | Inductive |
|  | Access to Primary Care | Patient ability to access secondary/more advanced care. | The private sector is not yet well established in this under-developed area. But since after the coup, as more doctors came into the area, the health services provided by medical doctors are became available here, in the form of charity clinic or a private clinic. MAM is providing NCD healthcare services in some villages in Kyainseikkyi township with their mobile medical team, but they are still not able to cover a large population. There are pharmacy stores which provides BP and glucose measurements. Some large grocery stores are also selling medicines like paracetamol, anti-hypertension and diabetes drugs. | Deductive |
|  | Access to Secondary Care | Patient ability to access secondary/more advanced care. | Some VTHCs are closer to a government hospital than to a KDHW hospital, and they were able refer patients to the nearest government hospital to avoid the long trip. Before the coup, the cooperation between EHOs (Ethnic Health Organizations), VTHCs, and the government MoHS hospitals was strong, but it has been severely disrupted since the military coup. | Deductive |
|  | Access to laboratory studies | Patient access to blood testing. | In terms of the lab, advanced and basic should be separated. As I previously stated, three or four locations have advanced labs, such as lab technicians. They keep the lab technician. However, there are rest places where basic lab services are available. | Deductive |
|  | Longitudinal health Information Systems | Ability to maintain longitudinal patient records | demographic health survey, the one we look at, is outdated already in 2014 and although the new one is planned before the coup, now is postponed and keep postponing. So, we are quite sure that the disease, the mortality rates are increasing, especially in this context. So, although there is no concrete statistic, up-to-date statistic, we, we are quite sure that we still need to focus on these and maternal and child health, which has been the problem already in this region. | Deductive |
|  | Referral related | Referral challenges, transportations and cost | Mostly, the patients are poor, not wealthy. So, if she or he has to give certain amount of money to go to the clinic, some will deny, even if it's in life danger. They will say we don’t have money, we don't have a bike, we can’t rent a car to go to the clinic. That's why I want this, the referral, bring from home to a clinic. That, it will be helpful a lot. | Inductive |
|  | Suggestions | Suggestions by interviewees | in next two or three years, while we improve our treatments for communicable diseases, we should also focus on non-communicable diseases as they need long-term cares. | Inductive |
| Donor | Conflict | Any quotes related to conflict/increased conflict. | Because of the political conflict, these patients cannot come to us, so they are among of suffering from the affected. Next, the patients who are living inside Myanmar, they are more suffering from this affected. | Deductive |
|  | Funding for CVD Risk Management | Discussion of funding for CVD risk management | I wish that there are more fundings coming for this NCDs especially on CVD, that’s definitely needed and even for cancer I have a research by myself, just informal way,........So I really wish more fund is coming and also based on your research, we can find more Partners and raise more funds. | Deductive |
|  | Funding for Primary Health System Strengthening | Quotes relating to donor interest in primary care HSS | ...ethnic health system strengthening group, along the Eastern Burma border. They have their long vision and this is how our access to health fund, we are also trying to support. What do you need, what do you want on next five years and then we will follow. | Deductive |
|  | Perceived burden of CVD Risk | Quotes relating to perception of CVD risk as a burden of disease in the catchment area. | If we say high blood pressure and diabetes, we can say why these diseases are increasing. We have a question like that. However, our priorities are diarrhea and pneumonia, and they are the common ones. That is why we give priority to treatment if it is a common disease, we prioritize more. Ah… if it is heart disease, it is not a priority. Yet, if they come, we provide the service, like transferring patients but there is no service like we do follow-up for ANC patients. | Deductive |
|  | CDM Doctors | Discussion of CDM doctors impact on EHO health system | Because of the coup, we get a lot of CDM doctors and they can give both prevention and the treatment for CVD. | Deductive |
| Excluded codes | Medication Safety | Any concerns about administering CVD medications | Covered by other codes | Deductive |
|  | Secondary care emphasis | Discussion of EHO priorities on secondary care. |  | Deductive |
|  | VHW role | Any discussion of what VHWs currently do or might do related to CVD care |  | Deductive |
|  | Capacity of Clinics | ability of clinics to meet needs for CVD risk management |  | Deductive |
