# Supplementary material for: Development of a village health worker care model to reduce cardiovascular risk in areas of armed conflict: Qualitative results from a participatory planning process in Myanmar
Source: PLOS Glob Public Health. 2026 Nov 6;6(11):e0005610. doi: 10.1371/journal.pgph.0005610 (PMC13743020; doi:10.1371/journal.pgph.0005610)
Supplement: Supplementary file 3 [file pgph.0005610.s003.docx]

**S1 Text: Qualitative Interview Guide, EHO Leadership, Program Managers**

 I. Introduction/Consent

*Hello and thank you for talking with me today. My name is _________________ and I am a health researcher working with CPI. I am interested in learning about the diagnosis and treatment of chronic diseases in the Ethnic Health Organization (EHO) clinics in Eastern Burma. We are meeting with EHO experts and stakeholders to understand the status of cardiovascular disease primary and secondary prevention, and we hope that this information will help strengthen the delivery of those services in that community. Cardiovascular diseases include things like strokes and heart attacks, and risk factors for cardiovascular disease include diabetes, high blood pressure, smoking, betel nut use, obesity and a lack of exercise (among others).  You will not receive any money for being here, and your participation will not affect any relationships with CPI or other organizations, funding to your organization, or any other benefits.*

*Everything you say here is completely confidential. We will not use your name or tell anyone you were here.  You are free to leave at any time, and your participation is completely voluntary. We are interested in your experiences as a health worker in the community.*

*This discussion will last no more than 1 hour. Our conversation will be recorded, so please do not use your name or names of individuals, and do not mention any information that can identify you or them. The recordings will be transcribed and then destroyed to protect your privacy. Researchers will analyze what you have said to understand how people access services for chronic diseases at EHO clinics, and how these services can be improved. This information may be shared in a general, non-identifiable way, with interested agencies and other colleagues. We will also have a short report for you that summarizes some of our learning and everyone’s recommendations.*

*Thank you again for your time. If you have any questions after the end of our discussion, please contact  Parveen Parmar* [*pparmar@usc.edu*](mailto:pparmar@usc.edu)*.*

II. KI Questions

1. What are the top priorities of your health system in the next 1-3 years?
2. How does prevention/treatment of cardiovascular disease rank among these priorities  (treatment of individuals with hypertension, diabetes, or history of heart attack or stroke)?
3. How do you envision integrating these priorities? What is your vision for a comprehensive, horizontally integrated primary health care system? (Prompt: how can we avoid multiple isolated vertical programs?)
4. Tell me about prevention and treatment of cardiovascular disease within your EHO health system. How do patients with cardiovascular disease access care? What are strengths of this system, what are weaknesses or potential problems with this system?
   a. Which facilities (clinics, hospitals)
   b. village-level services
   c. how do they interact?
   d. how are they staffed
      i. What are their roles now?
      ii. How are they paid (salary, stipend, etc.)?
      iii. CDM doctors–what role do they play?
   b. Which donors support care for CVD?
   c. health information
   d. Supply chain (medications, other?)

5. Are there any programs you’re aware of that specifically target cardiovascular risk or risk factors in your EHO catchment areas? Please tell  us about these.

6. Now I would like to ask you about challenges and successes faced by current programs to reduce cardiovascular risk. Many health systems in many countries have a difficult time identifying all individuals with cardiovascular risk factors, and achieving treatment targets for those risk factors. For example, previous studies suggest that only about half of patients with high blood pressure in the United States have achieved control of their blood pressure; the rate of controlled hypertension in Myanmar may be 10% or less.
   a. What is working well with regards to your current system (screening for disease, linkage to care, medications, referrals, education, etc.)?
      i. What advantages does your EHO have in delivering these services, compared to other health systems and providers?
         1. Prompt: trust, language, culturally sensitivity, other.
      ii. What are the reasons for these successes?
   b. What could be improved with regards to the current system (above factors) and how?
      i. Prompts: Human resources; financial resources; knowledge/expertise; competing priorities, supply chain, security, quality of care, Coverage, equity
   b. How does the referral system from clinics to higher levels of care work? What works well and what could be improved?
   c. Describe successes and challenges securing funding for CVD care, such as grants.
   d. What role, if any, can village health workers (VHWs) play in care for those living with cardiovascular disease and/or risks?

7. How has the current political situation/conflict affected cardiovascular disease services? How has the system changed since the coup?
   a. Prompts:
      i. relationships with other stakeholders
      ii. organizational priorities
      iii. donor priorities
      iv. service delivery models, staffing, supply chain, etc.
          1. mobile clinics
   b. Are there creative approaches that would help to increase coverage and maintain adherence in the current unstable situation?
      i. What are your thoughts on multi-month dosing? (providing 2 or 3 months of pills, in case there is a disruption in services or individuals are forced to move)

8. How do you determine where to locate clinical services in relation to population/burden of disease? For example, do you have any maps of the areas served by your clinics with surrounding villages, transportation times, geography, etc.?

9. The current plan for research (which is flexible!) may test a village level intervention to improve screening and linkage to care for people with hypertension, diabetes, and other risks for cardiovascular disease.  This would involve a village health worker going from home to home to test everyone over 40 for diabetes, high blood pressure, obesity, and to ask them if they smoke.  People with risks for cardiovascular disease would then be referred to the clinics for care.
   a. How might an intervention like this work best?  One VHW in each village? One VHW to cover multiple villages?  Could this be a part of an existing VHW role? Periodic outreach by clinic based CHWs?
   b. What type of skills might this worker need?
      i. health related
      ii. navigating complex physical and political situation
   b. Evidence shows that village level health workers work best when paid.  How might payment work? What are the limitations?
   c. How might this village level worker help with resilience to conflict? (medication stores, follow displaced villagers to a new location etc.)

10. Now that you have heard this information about this draft research plan, what if any ideas do you have to modify and improve this research? [*if they haven’t heard about the research, please tell them].*
    a. we welcome your suggestions on how to make the research and findings useful to your organization and the people living with CVD risk factors in the target area

III. Closing

Those are all the questions I have for today.

1. Is there anything else you would like to add?

Thank you for taking the time to answer our questions. If you think of anything else that you feel may be important or have any other questions or concerns, don’t hesitate to contact us.
