# Supplementary material for: Development of a village health worker care model to reduce cardiovascular risk in areas of armed conflict: Qualitative results from a participatory planning process in Myanmar
Source: PLOS Glob Public Health. 2026 Nov 6;6(11):e0005610. doi: 10.1371/journal.pgph.0005610 (PMC13743020; doi:10.1371/journal.pgph.0005610)
Supplement: Supplementary file 4 [file pgph.0005610.s004.docx]

**S2 Text:  EHO Clinic Leadership Qualitative Interview Guide and Facility Assessment Tool.**

*Hello and thank you for talking with me today. My name is _________________ and I am a health researcher working with CPI. I am interested in learning about the diagnosis and treatment of chronic diseases in the Ethnic Health Organization (EHO) clinics in Eastern Burma. We are meeting with EHO experts and stakeholders to understand the status of cardiovascular disease primary and secondary prevention, and we hope that this information will help strengthen the delivery of those services in that community. Cardiovascular diseases include things like strokes and heart attacks, and risk factors for cardiovascular disease include diabetes, high blood pressure, smoking, betel nut use, obesity and a lack of exercise (among others).  We will be asking about the cardiovascular disease among adults only. You will not receive any money for being here, and your participation will not affect any relationships with CPI or other organizations, funding to your organization, or any other benefits.  We will also not share what you said with your employers or leadership in a way that will identify who you are.*

*Everything you say here is completely confidential. We will not use your name or tell anyone you were here.  You are free to leave at any time, and your participation is completely voluntary. We are interested in your experiences as a health worker in the community.*

*This discussion will last no more than 2 hours. We will also conduct a facility survey, we have permission from your leadership to do so. Our conversation will be recorded, so please do not use your name or names of individuals, and do not mention any information that can identify you or them. The recordings will be transcribed and then destroyed to protect your privacy. Researchers will analyze what you have said to understand how people access services for chronic diseases at EHO clinics, and your perspectives on how these services can be improved. This information may be shared in a general, non-identifiable way, with interested agencies and other colleagues. We will also have a short report for you that summarizes some of our learning and everyone’s recommendations.*

*Thank you again for your time. If you have any questions after the end of our discussion, please contact  Parveen Parmar pparmar@usc.edu.*

1. Can you describe the services provided by this clinic?
   a. Malaria, TB, trauma, CVD, etc.  What labs if any are available?  What other supplies are available (sphygmomanometer, glucometer, etc.?)
   b. WHo are the providers at this facility? What do each of these provider types do? What type of training do each of these providers have?
   c. Which of these providers are involved in the care of patients with risks for cardiovascular disease? What do they do for these patients?

2. Can you describe the health services provided in villages served by this clinic?
   a. Prompts: VHWs for all programs (malaria, SRH etc), screening, education, etc.  Which villages are targeted, how often, etc?
   b. ***[Show Clinic Leadership SPP village and clinic map]***.  Here is a map of the villages in this clinic’s catchment area, transportation times, etc.  Can you look at it and let us know if it is correct?  Please edit it as necessary.
      i. *If an SPP map DOES NOT exist yet, please draw a  map that includes the clinic, the surrounding villages form which patients come, transportation times, barriers in rainy and dry season, costs for transport if any, etc.*

3. How are medical records kept, and what information is recorded? Who is this shared with and how/how often? How is this information connected to VHWs/patients in the community, if at all?
   a. Prompts: paper vs. electronic; software.
   b. May we have a blank copy (on paper or electronically) of any medical record keeping systems? [*take a photo or use a USB if needed].*

4. Can you tell me about the main factors that contribute to increased risk for heart attack and stroke among this clinic’s population (diet, obesity, lack of exercise, alcohol use, smoking (tobacco), betel quid chewing?  Hypertension, diabetes, prior history of stroke or heart attack?)?
   a. Approximately how many adults have hypertension, diabetes, and other risks for cardiovascular disease?
   b. Approximately what percentage of these  patients do you think are getting treatment?

5. Tell me the story of how someone with hypertension, diabetes, history of stroke or heart attack accesses care at the clinic, step by step.
   a. Whom do they see first? Which staff do they see, and what do these staff do?
   b. What exams are done?
   c. What if any laboratory tests are done?
      i. Are there laboratory tests available nearby?
      ii. What do these cost?
      iii. Are these accessible, and why or why not?
   b. WHat data is recorded and where?
      i. (height/weight, blood pressure, glucose etc.)
   b. How do they get medications?
      i. duration of prescription (2 weeks, 1 months etc)
      ii. any issues with supply chains?
   b. What if any education do they receive?
   c. What other services do they get?
   d. How often are they asked to come back? How often do they come back?
   e. If they miss an appointment or do not refill their prescription, what is the process for following up with them? Who is in charge of this process etc
   f. What role, if any, do VHWs play?

6. What, if any, other facilities or health providers provide care for individuals with cardiovascular disease and/or risk factors for cardiovascular disease in this clinic’s catchment area? [traditional healers, others?]

7. How do staff at this clinic feel treating cardiovascular disease? Do they feel they have enough training? Supplies? Ability to identify important conditions?
   a. What would improve clinic/clinic staff readiness to treat cardiovascular disease risks like hypertension and smoking?
      i. Do you prescribe antihypertensives? Do you feel this is something you should do, and why or why not? Would you like more training and if so on what specifically?
      ii. Do you prescribe statins? Do you feel this is something you should do, and why or why not? Would you like more training and if so on what specifically?
      iii. Do you prescribe aspirin? Do you feel this is something you should do, and why or why not? Would you like more training and if so on what specifically?

b. How would this clinic ideally like to be involved in improving care for people living with cardiovascular disease risk?

8. Tell me about the clinical guidelines and other protocols that are used at this clinic to help with diagnosis and treatment
   i. Prompt: Burma Border Guidelines; WHO PEN protocol; WHO risk charts… other?
   ii. Tell me about the use of cardiovascular risk charts or other cardiovascular risk equations / calculators?

9. Tell me about the referral process for cardiovascular conditions.
   a. Prompts:
      i. Acute conditions / emergencies: stroke, heart attack, acute edema or shortness of breath (eg congestive heart failure exacerbation)
      ii. Type of facility and location (where do they go?)
      iii. Transportation/ambulance (how do they get there)
      iv. Payment / reimbursement
      v. Acceptance at referral facility (are referred patients treated with respect?)
      vi. What are the costs associated with referrals (out of pocket, pooled funds etc)
      vii. chronic conditions: diabetes, hypertension, stroke, heart attack, (heart failure)…
      viii. Impact of the coup (have referral options changed?)
      ix. What are the challenges with the referral system? What is working well? What are the opportunities to improve referrals?

10. Now I would like to ask you about challenges and successes faced by current programs to control hypertension and other risk factors for heart attack and stroke. Health systems in many countries have a difficult time identifying all individuals with cardiovascular risk factors, and achieving treatment targets for those risk factors. For example, previous studies suggest that only about half of patients with high blood pressure in the United States have achieved control of their blood pressure; EBRMS data shows only a very small percentage of patients with hypertension and risks for cardiovascular disease come to the clinic, and return for medications and care regularly.
    a. What is working well at this clinic? (screening for disease, medications, referrals, education, etc.)? What are the reasons for these successes?
    b. What could be improved with regards to the current system (low utilization and return visits) and how?
       i. Prompts: Human resources; funding; patient knowledge, staff knowledge; competing priorities, supply chain, security, etc.
    b. How might [EHO] leadership support improved CVD service delivery at this clinic?

11. How might VHWs improve care for those living with cardiovascular disease and/or risks, if at all?

12. How has the current political situation/conflict affected cardiovascular disease services at this clinic?
    a. How has the system changed?
    b. How can CVD care be made more resilient to periods of insecurity?
a. Prompts
   i. How might protocols be simplified?
   ii. Is it feasible to provide more doses of medication (for example, 2 or 3 months of pills instead of 1 month)
   iii. How to ensure individuals have blood pressure controlled, quit smoking, take a statin?
   iv. Other ideas

13. What do you think most EHO patients think about cardiovascular disease?
    a. Prompts:
       i. Do they perceive cardiovascular risk factors as a priority? Why or why not?
       ii. How do you think they know when to seek care?
       iii. How do they approach the idea of taking a medication every day, possibly for the rest of their lives?
       iv. How often should patients come back, and do they come back? Why or why not?

III. Closing

Those are all the questions I have for today.

1. Is there anything else you would like to add?

Thank you for taking the time to answer our questions. If you think of anything else that you feel may be important or have any other questions or concerns, don’t hesitate to contact us.
