# Supplementary material for: Development of a village health worker care model to reduce cardiovascular risk in areas of armed conflict: Qualitative results from a participatory planning process in Myanmar
Source: PLOS Glob Public Health. 2026 Nov 6;6(11):e0005610. doi: 10.1371/journal.pgph.0005610 (PMC13743020; doi:10.1371/journal.pgph.0005610)
Supplement: Supplementary file 5 [file pgph.0005610.s005.docx]

**S3 Text:  Donor/NGO Representative Qualitative Interview Guide**

**I. Introduction/Consent**

*Hello and thank you for talking with me today. My name is _________________ and I am a health researcher from _______________. I am interested in learning about the diagnosis and treatment of chronic diseases in the Ethnic Health Organization (EHO) clinics in Eastern Burma. We are meeting with donors, EHO experts and stakeholders to understand regional funding. You will not receive any money for being here, and your participation will not affect any of your interactions with CPI or other affiliated agencies.*

*Everything you say here is completely confidential. We will not use your name or tell anyone you were here.  You are free to leave at any time, and your participation is completely voluntary. We are interested in your perspectives as representative of a donor agency and as a regional expert.*

*This discussion will last no more than 1 hour. Our conversation will be recorded, so please do not use your name or names of patients. Please do not mention any information that can identify you or others. The recordings will be transcribed and then destroyed to protect your privacy. This information may be shared in a general, non-identifiable way, with interested agencies and other colleagues. We will also have a short report for you that summarizes some of our learning and everyone’s recommendations.*

*Thank you again for your time. If you have any questions after the end of our discussion, please contact _______.*

**II.  Questions**

1. What are the main funding priorities for your organization in Eastern Myanmar? For organizations who also fund work in this region? How do you envision these changing, if at all, over the next few years?

2. Can you tell me about your and other regional organizations’ history funding for cardiovascular disease interventions? How do you see this changing moving forward, if at all?

3. What is your sense of where needs are currently being met with regards to cardiovascular disease programming, and where needs still exist, in this region?
   a. Prompts: Underserved regions, primary care, referral networks, stable funding, M&E….

4. What research is needed (if any) to strengthen delivery of cardiovascular disease care in this region?

5. How has evidence of national and regional disease burden informed the grantmaking priorities of your institution?
   a. Prompt: The Global Burden of Disease Study / IHME estimate that stroke and ischemic heart disease are the leading causes of death in Myanmar. In Eastern Myanmar specifically, a high quality population-based survey conducted in 2019 demonstrated that approximately half of adults over 40 years old have modifiable CVD risk factors.
   b. How has information on the burden of CVD in Myanmar influenced the priorities of your institution?

6. How do Humanitarian Standards such as SPHERE, the priorities outlined in the Humanitarian Response Plan for Myanmar, and country-specific health service priorities inform the grantmaking priorities of your institution?

a. Prompt: SPHERE outlines Minimum Standards for humanitarian emergencies; SPHERE standards now extend to NCDs in general and CVD risk factors such as hypertension and diabetes in particular. Clinical guidance for CVD risk factors in humanitarian emergencies is available in the humanitarian PEN documents developed by WHO and IRC. The Humanitarian Response Plan for Myanmar in 2022 (and 2023?) explicitly endorses access to NCD care as a key priority for the health cluster. The Ministry of Health and Sports (MOHS) of Myanmar includes CVD as part of its basic package of health services and this basic package has been adopted by most ethnic and community based services providers in Eastern Myanmar.

7. How important are cost-effectiveness and equity to setting funding priorities at your institution?
   a. how does the organization go about assessing and prioritizing equity-efficiency tradeoffs?

8. How important is it to the priority-setting process at your institution to hear from local implementing partners and community members about their funding priorities and funding gaps?
   a. Prompt: Funding for CVD services in Eastern Myanmar is severely limited; few health organizations are successful in raising funds for CVD programs.

9. What additional information would be useful as your institution considers funding services that address CVD?

10. We are conducting a cluster randomized trial that documented the feasibility, cost-effectiveness and equity impacts of a CVD service model in remote rural areas of Eastern Myanmar. How might your organization use results of such a trial?

a. What specifically would you be looking for in such a study?
b. We welcome any specific ideas you have of what to measure and how to report results…

Final questions:

11. Who in your opinion should be accountable for ensuring Humanitarian Standards and stated priorities for CVD services are achieved (or even attempted) in emergencies in general and in Eastern Myanmar in particular?

12. Which other donors are in a position to contribute to CVD services? Whom else might we interview?

**III. Closing**

Those are all the questions I have for today.

1. Is there anything else you would like to add?

Thank you for taking the time to answer our questions. If you think of anything else that you feel may be important or have any other questions or concerns, don’t hesitate to contact us.
