# Supplementary material for: Development of a village health worker care model to reduce cardiovascular risk in areas of armed conflict: Qualitative results from a participatory planning process in Myanmar
Source: PLOS Glob Public Health. 2026 Nov 6;6(11):e0005610. doi: 10.1371/journal.pgph.0005610 (PMC13743020; doi:10.1371/journal.pgph.0005610)
Supplement: Supplementary file 6 [file pgph.0005610.s006.docx]

**S4 Text: Qualitative Guide: Patient/VHW Qualitative Interview Guide**

**I. Introduction/Consent**

*Hello and thank you for talking with me today. My name is _________________ and I am a health researcher from _______________. I am interested in learning about the diagnosis and treatment of chronic diseases in the Ethnic Health Organization (EHO) clinics in Eastern Burma. We are meeting with patients affected by cardiovascular disease or who have risks for cardiovascular disease to understand their situation. Cardiovascular diseases include things like strokes and heart attacks, and risk factors for cardiovascular disease include diabetes, high blood pressure, smoking, betel nut use, obesity and a lack of exercise (among others).  You will not receive any money for being here, and your participation will not affect the services you recieve or any services received by your community.*

*Everything you say here is completely confidential. We will not use your name or tell anyone you were here.  You are free to leave at any time, and your participation is completely voluntary. We are interested in your perspectives as a patient, and we will do our best to ensure your knowledge is used to improve services in this region.*

*This discussion will last no more than 1 hour. Our conversation will be recorded, so please do not use your name or names of patients. Please do not mention any information that can identify you or others. The recordings will be transcribed and then destroyed to protect your privacy. This information may be shared in a general, non-identifiable way, with interested agencies and other colleagues. We will also have a short report for you that summarizes some of our learning and everyone’s recommendations.*

*Thank you again for your time. If you have any questions after the end of our discussion, please contact _______.*

**II.  Questions**

1. Based on your experience as a patient and the experiences of people you know in your community, please describe how patients living in Karen State with risk factors for cardiovascular disease (high blood pressure, diabetes, smoking, etc.)  access care here in this region. [Prompts: clinics, pharmacies, CHVs, traditional healers, specialists, medications, testing].

2. Thinking about the need for chronic care for these diseases mentioned above, what works well, and what does not work well?

3. What are all of the ways you get care for your chronic diseases like high blood pressure/diabetes or high cholesterol? (clinic, hospital, traditional healer, pharmacist, etc).

4. When have you been referred to for care of high blood pressure/diabetes/heart attacks or strokes, if at all, and where were you sent? What was your experience like there?

5. How could this system be improved, if at all?  [Prompts: primary/specialist care, stable access to medications, testing, education, diet, security…?]

6. Please describe your experience and the experiences of your community in working with village health workers. [Prompts: Tell me the story of what they do, a good one and a bad one].  What are their strengths, and what are their weaknesses?

7. How could access to care for high blood pressure and diabetes (screening, medications, clinic visits, education, etc.) be improved, if at all?

8. What are the main problems you and patients like you in this community face in accessing health care in this setting continuously? [Prompts: Primary care, medications, referrals, transportation, secondary/tertiary care, security, current political situation/conflict….?]

a. How often do you go to the clinic for check ups, medication, etc?
b. Is it difficult to go to the clinic, and if so why?
c. What is your experience like at this clinic?  We will keep your answers confidential.
d. How has conflict affected access to health care for cardiovascular disease in this region? Referrals?

9. Can VHWs play a larger role in screening/treatment/education on cardiovascular disease, and if so how?
9. What from your perspective are the biggest needs for this community with regards to health? Where do conditions like high blood pressure and diabetes fall into this?

**III. Closing**

Those are all the questions I have for today.

1. Is there anything else you would like to add?

Thank you for taking the time to answer our questions. If you think of anything else that you feel may be important or have any other questions or concerns, don’t hesitate to contact us.
